# Acute and Chronic Effects of a High-Intensity Interval Training Shock Microcycle on Cell-Free DNA: A Randomized Controlled Trial

**DOI:** 10.1186/s40798-025-00923-9

**Published:** 2025-11-21

**Authors:** Aleksandar Tomaskovic, Tilmann Strepp, Thomas Leonhard Stöggl, Elmo W. I. Neuberger, Perikles Simon, Nils Haller

**Affiliations:** 1 Department of Sports Medicine, Rehabilitation and Disease Prevention, Johannes Gutenberg University of Mainz, Mainz, Germany; 2 Department of Sport and Exercise Science, University of Salzburg, Salzburg, Austria; 3 Red Bull Athlete Performance Center, Salzburg, Austria; 4 Division of Exercise and Movement Science, Institute for Sport Science, University of Göttingen, Göttingen, Germany

**Keywords:** Exercise immunology, Load monitoring, Liquid biopsy, Blood biomarker, Neutrophil extracellular traps (NETs), Muscle injury

## Abstract

**Background:**

This study aimed to evaluate acute and chronic exercise-induced changes in cell-free DNA (cfDNA) concentrations during a 7-day high-intensity interval training (HIIT) shock microcycle in trained endurance athletes. Thirty-five participants were randomly assigned to one of three groups: a HIIT-only group (HSM), a HIIT plus low-intensity training group (HSM + LIT), and a control group maintaining regular training. The intervention included 10 HIIT sessions (5 × 4 min at 90–95% maximum heart rate) over 7 days, with HSM + LIT completing an additional 30 min of low-intensity training after each session. Physiological exercise testing (PET) was conducted at baseline, 3-, 7-, and 14-days post-intervention. On days 2 and 7 during the intervention, HIIT sessions were supervised in both morning and afternoon, and venous blood samples were collected at rest, immediately post-exercise, and 30 min post-exercise to measure cfDNA for 90 and 222 bp fragments. Correlations between cfDNA and physiological exercise variables such as peak power output (PPO), running velocity at lactate threshold (LT), and VO₂_max_ were analyzed.

**Results:**

cfDNA^90^ (10.4-fold, *p* < 0.001) and cfDNA^222^ (12.4-fold, *p* < 0.001) increased significantly after PET. In addition, cfDNA^90^ (17.1-fold, *p* < 0.001) and cfDNA^222^ (20.2-fold, *p* < 0.001) increased after HIIT, both remaining significantly elevated 30 min post-HIIT (both *p* < 0.001). cfDNA^90^ concentrations were higher in afternoon (22.4-fold) compared to morning HIIT sessions (17.2-fold, *p* < 0.001). A significant interaction effect was found between group and measurement point for cfDNA^90^ (*p* < 0.001) and cfDNA^222^ (*p* < 0.001), with higher concentrations in HSM + LIT compared to HSM 30 min post-HIIT. cfDNA^90^ showed moderate correlations with PPO (*r* = 0.48, *p* < 0.001), LT (*r* = 0.36, *p* < 0.001) and VO_₂max_ (*r* = 0.30, *p* = 0.01). cfDNA^222^ correlated moderately with VO_₂max_ (*r* = 0.34, *p* = 0.001) and slightly with PPO (*r* = 0.21, *p* = 0.05). No chronic changes in cfDNA were observed throughout the study period.

**Conclusions:**

cfDNA is a reliable marker for detecting acute exercise-induced stress. However, the potential of cfDNA for detecting chronic adaptations in short-term, high-intensity interval training settings, such as a HIIT shock cycle, appears limited thus far.

*Trial registration* clinicaltrials.gov, NCT05067426. Registered 05 October 2021—Retrospectively registered, https://clinicaltrials.gov/ct2/show/NCT05067426.

**Graphical Abstract:**

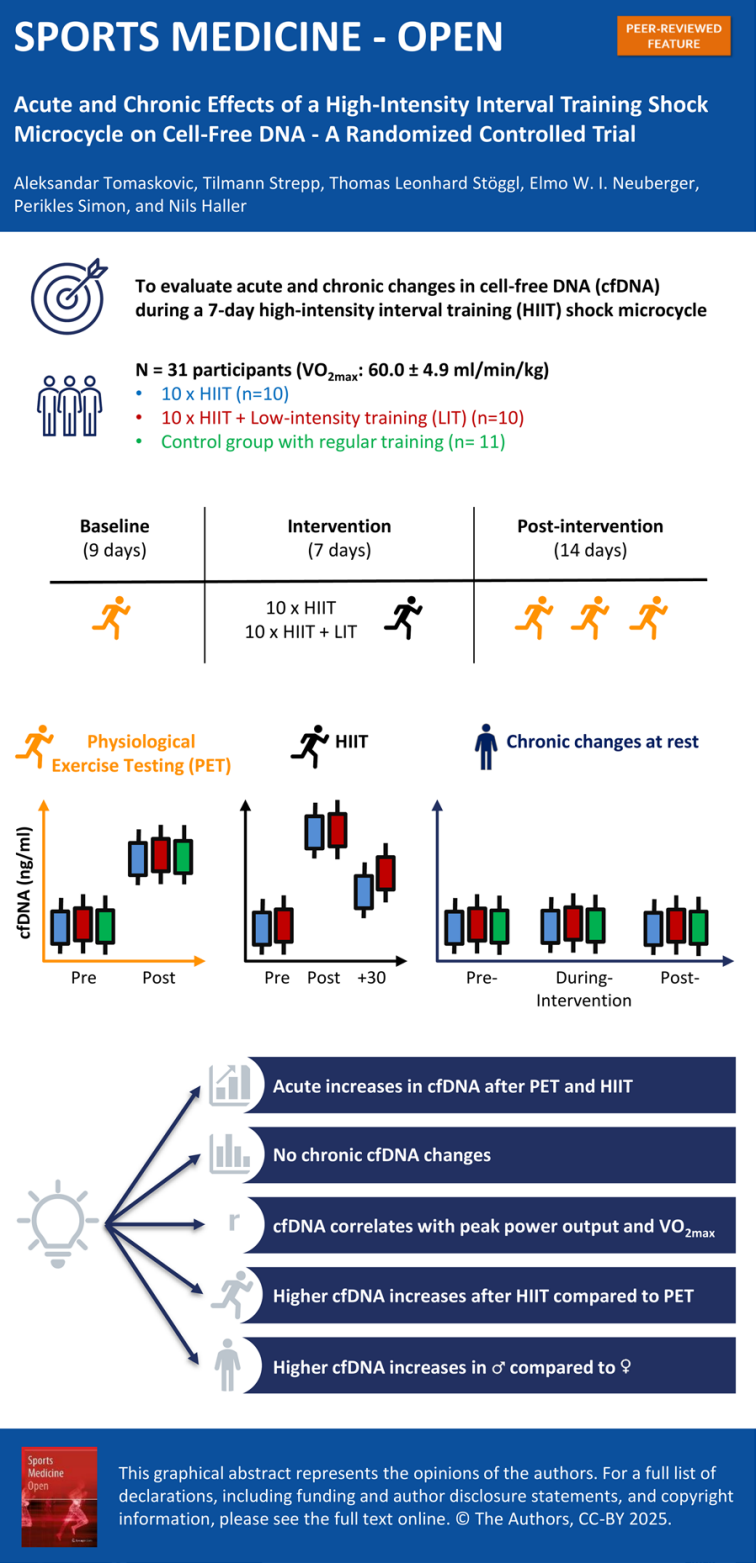

**Supplementary Information:**

The online version contains supplementary material available at 10.1186/s40798-025-00923-9.

## Key Points

- Acute increases in cfDNA concentrations were observed after physiological exercise testing and high-intensity interval training (HIIT).
- No chronic cfDNA changes were observed during and after a HIIT shock microcycle.
- Acute increases in cfDNA concentrations correlated with physiological exercise variables e.g. peak power output, running velocity at lactate threshold and VO_2max_ during acute exercise.
- Higher increases in cfDNA were found after HIIT compared to physiological exercise testing.
- Higher increases in cfDNA were found after HIIT in the afternoon compared to morning.
- Higher increases in cfDNA were observed in males compared to females.

**Supplementary Information:**

The online version contains supplementary material available at 10.1186/s40798-025-00923-9.

## Background

Training load monitoring is a fundamental aspect of daily sport science practice to assess the training response, minimize injury risk, and optimize performance [1, 2]. The search for valid tools to monitor the external (i.e., the physical work, measured through e.g. distance covered, power output) and internal training load (i.e., the psychophysiological response) employing methods like heart rate (HR), questionnaires, performance testing, or blood biomarkers, is continually expanding [2]. Internal training load monitoring tools should demonstrate sensitivity to exercise load ideally not confounded by other factors such as diet or circadian rhythm [3]. In addition, markers should be easily and frequently measurable, ensuring prompt availability of results. Finally, the measurement process should seamlessly integrate with the ongoing training regimen [4, 5]. As of yet, no marker meets all criteria, and there is a lack of consensus on the essential variables for monitoring both external and internal load.

With respect to the internal load, practitioners commonly rely on easy-to-use tools such as HR or questionnaires as valid tools to monitor acute and chronic changes in training load [1, 6]. HR is a useful indicator of the internal load in endurance sports, but it is less reliable in intermittent sports or resistance training. Additionally, daily fluctuations in HR can complicate the interpretation of the data [1, 6]. Questionnaires harbor the inherent risk of manipulation, influenced by social desirability and the pressure to perform. Additionally, there is a potential to either over or underestimate the workload using questionnaires [7–9]. Thus, a combination of subjective and objective tools, such as blood-based biomarkers [3], is warranted. Established biomarkers such as lactate or creatine kinase have often been used to monitor aspects such as the acute training load or muscle damage [10, 11]. However, there is no single blood marker or a combination of markers that effectively reflect objective training load or, more importantly, demonstrate a consistent relationship with changes in performance or injury risk [3].

In this respect, circulating cell-free DNA (cfDNA) has gained attention as a biomarker for monitoring internal training load [12, 13]. Previous research has demonstrated that cfDNA responses to incremental exercise are consistent and reproducible, showing low intra-individual variability and strong correlations with established physiological markers such as HR and lactate concentrations, thereby supporting its reliability as a biomarker in exercise settings [14]. cfDNA can be quantified using assays that target fragments of various base pair (bp) lengths, including tissue-specific fragments, potentially enabling personalized exercise prescriptions [15]. Exercise-induced increases in cfDNA concentrations originate primarily from cells of the hematopoietic lineage, with a biological half-life time of around 15 min [16]. Cell-type-specific epigenetic analysis has revealed that a significant portion of cfDNA released during exercise is derived from neutrophil granulocytes, rapidly released within minutes through active secretion via neutrophil extracellular traps (NETs) [17, 18]. Previous exercise studies have targeted different fragment sizes to explore the kinetics of cfDNA during exercise [17, 19, 20], following the idea that both short and long cfDNA fragments provide complementary insights. During acute exercise, cfDNA released via NETs is digested by deoxyribonuclease (DNase), generating short cfDNA fragments [17, 21]. In contrast, long cfDNA fragments are associated with processes like necrosis, indicative of cellular damage [20]. Therefore, studies examining the relationship between cfDNA fragment size, training intensity, and training volume are important for understanding the cfDNA response to exercise.

Acute cfDNA increases have been observed in response to short-term maximal exercise [14], prolonged aerobic exercise [17], intermittent exercise [22], and resistance training [23, 24]. The magnitude of cfDNA increases was reported to depend on both exercise intensity and duration [25]. Elevated cfDNA concentrations during and immediately post-exercise have also been associated with other blood-based biomarkers, such as cytokines and chemokines [22, 26]. While the acute exercise-induced cfDNA response is well-researched and consistent, the chronic exercise responses have been studied to a lesser extent [14] with only a limited number of studies that have measured cfDNA concentrations at rest throughout different training programs [13, 20, 24, 27]. For instance, Fatouros et al. [24] found that resting cfDNA concentrations peaked during periods of high training volume and declined as training intensity was reduced in a weightlifting training program over several weeks. In contrast, Tug et al. [20] reported no significant increases in resting cfDNA concentrations, except for a transient increase 2 days post-exercise, in either low- or high-intensity strength training programs. Additionally, Haller et al. [13] observed that resting cfDNA concentrations are influenced by training load in professional football players, while Gentles et al. [27] noted a chronic increase in cfDNA during a regular football season. Regardless of the training modality, the acute increases in cfDNA concentrations are much more pronounced than the chronic cfDNA increases.

To date, no study has explored both the acute and chronic effects of an intensified endurance training period, i.e., a high-intensity interval training (HIIT) shock cycle, on cfDNA in trained athletes. The present study consisted of a 9-day baseline period, a 7-day intervention period, and a 14-day post-intervention period. Repeated blood samples were collected at rest and post-exercise to assess whether cfDNA could serve as a biomarker for both acute and chronic training load during intensified training periods. We hypothesize that cfDNA concentrations will change (i) acutely during exercise, and (ii) chronically at rest over the course of the study period. Exploratory analyses will reveal possible correlations with established physiological exercise variables such as maximum oxygen uptake rate (VO_₂max_).

## Methods

### Experimental Setup

This study was registered at ClinicalTrials.gov (identifier: NCT05067426) and the study protocol was published by Stöggl et al. [28]. Ethical approval was granted by the local ethics committee of the University of Salzburg (GZ 02/2021), with all procedures complying with the standards of the Declaration of Helsinki.

**Fig. 1:**
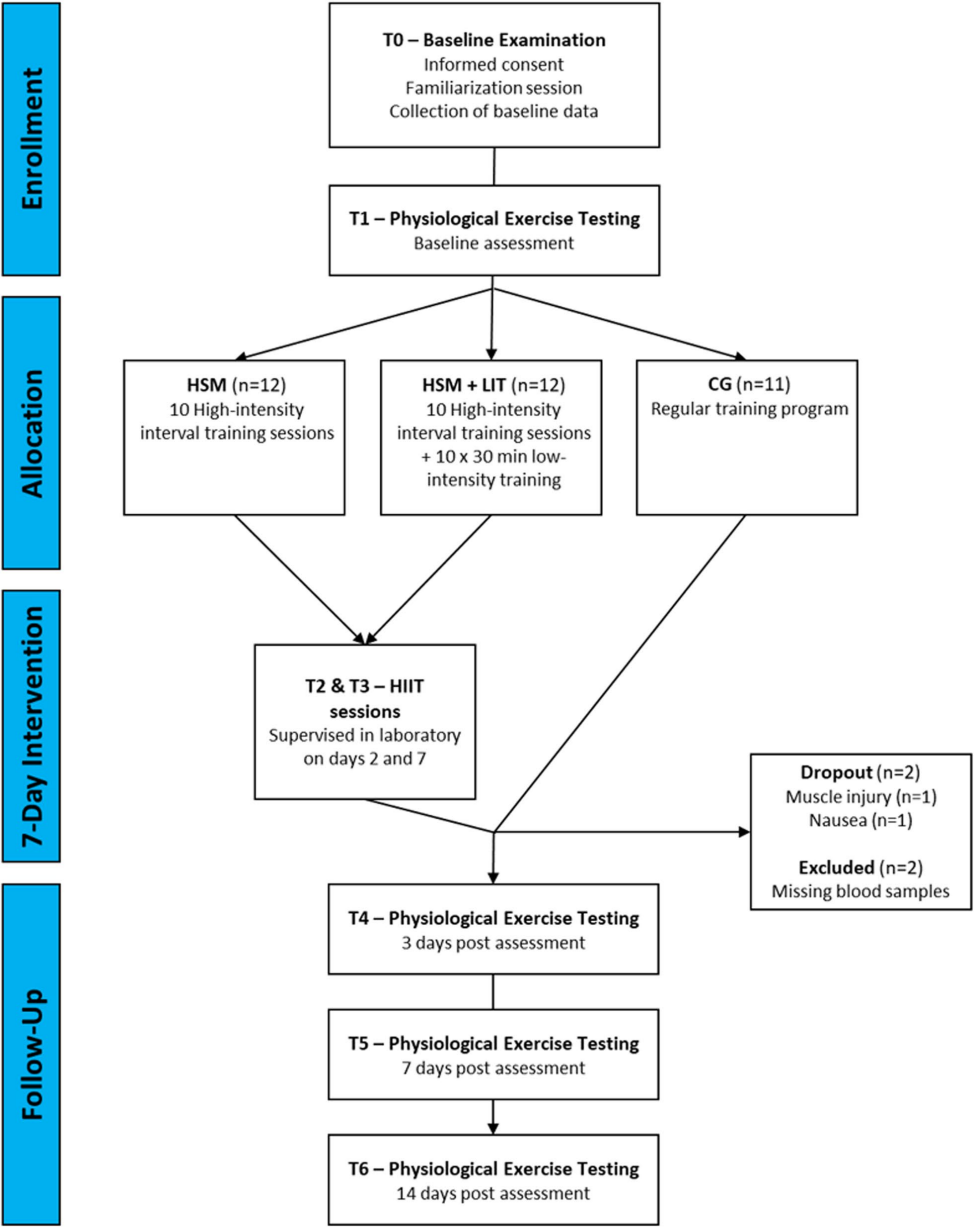
Study flowchart. The study was divided into a baseline period of 8–9 days (from T0 to T1), a 7-day intervention period, and a 14-day post-intervention period. Face-to-face appointments were at time points T0, T1, T4, T5, and T6 for all participants. The intervention groups completed high-intensity interval training (HIIT) sessions in the laboratory at T2 and T3. High-intensity interval training shock microcycle (HSM), HSM with additional low-intensity training (HSM+LIT) and control group (CG). Adapted from Stöggl et al. [28], with permission (licensed under CC BY 4.0)

Following a baseline period, 35 participants were randomly allocated to one of three groups: (i) intervention group 1 performed a 7-day HIIT shock microcycle with 10 HIIT sessions (HSM); (ii) intervention group 2 performed a 7-day HIIT shock cycle with 10 HIIT sessions including 30 min of low-intensity training (LIT) following each HIIT session (HSM + LIT); (iii) a control group (CG) continued with their regular training routine without any imposed restrictions or additional interventions. The study lasted four weeks for all participants, comprising an 8 to 9-day baseline period, a 7-day intervention period, and a 14-day post-intervention period. Figure 1 outlines the study flowchart.

During a baseline examination (T0), all participants provided informed consent and were familiarized with the study design and equipment. Regular training was continued during the baseline period, and participants underwent entry physiological exercise testing (PET) at time point T1 to determine eligibility and individual intensity ranges for HIIT sessions (if allocated to one of the intervention groups). The total duration of each PET session was approximately 35–38 min (including incremental stages, a standardized 8-minute rest period, and the subsequent ramp test). The following variables were measured: Rating of Perceived Exertion (RPE), maximum heart rate (HR_max_), maximum respiratory quotient (RQ_max_), VO_₂max_, peak capillary lactate concentration (La_peak_), peak power output (PPO), training impulse (TRIMP) [29], and running velocity at lactate threshold (LT). A list of all outcome measures can be found elsewhere [28].

During the post-intervention period, PET was repeated three times (T4 after 3 days, T5 after 7 days and T6 after 14 days) to assess the trajectories of performance changes [30]. Of the 10 HIIT sessions during the intervention period, four were monitored in a laboratory—two HIIT sessions at time point T2 (day 2 of intervention period) and two HIIT sessions at T3 (day 7 of intervention period). PET and HIIT sessions were conducted under controlled conditions by experienced exercise scientists at the University of Salzburg, Rif, Austria. Food and supplement consumption was not recorded, but participants were encouraged to maintain their usual diet. The CG followed their regular training routine and reported at time points T1, T4, T5 and T6.

## Participants

Out of a total of 43 recruited participants between February 2021 and December 2022, 8 participants did not meet the inclusion criteria, resulting in a sample size of 35 trained [11] endurance athletes. Eligible participants aged 18–45 years were required to have a VO_2max_ of ≥ 50 ml/kg/min (female) or ≥ 55 ml/kg/min (male). Alternatively, a 5 km time trial performance of ≤ 20:00 min (female) or ≤ 18:30 min (male) was sufficient to participate. Additional information on the inclusion criteria can be found in our study protocol [28].

## Intervention

Each participant was equipped with a Global Navigation Satellite System watch (Forerunner 935, Garmin, Kansas City, MO, USA), a HR chest strap (HRM Pro, Garmin, Kansas City, MO, USA), and a footpod (Wind v3, Stryd, Boulder, CO, USA) to measure running velocity, HR, and running power, respectively, during all outdoor and treadmill activities throughout the study period [28]. Throughout the 7-day intervention period, HSM and HSM + LIT participants conducted a total of 10 HIIT sessions. Each HIIT session consisted of 5 intervals of 4 min each, performed at an intensity of 90–95% of the individual HR_max_. Intervals were separated by a 2.5-min recovery period, incorporating 1 min of passive rest for blood sampling and 1.5 min of active recovery running at a velocity corresponding to a 1.5 mmol/l blood lactate concentration (La), determined at time point T1. Every session started with a standardized 10-min low-intensity warm-up program, resulting in a total session duration of 40 min. Compared to HSM, the HSM + LIT group underwent an additional 30 min of LIT following each HIIT session. This resulted in an extra LIT volume of 300 min, representing a 75% increase in the overall training volume. LIT was conducted at a velocity corresponding to a 1.5 mmol/l La as determined at T1.

**Fig. 2:**
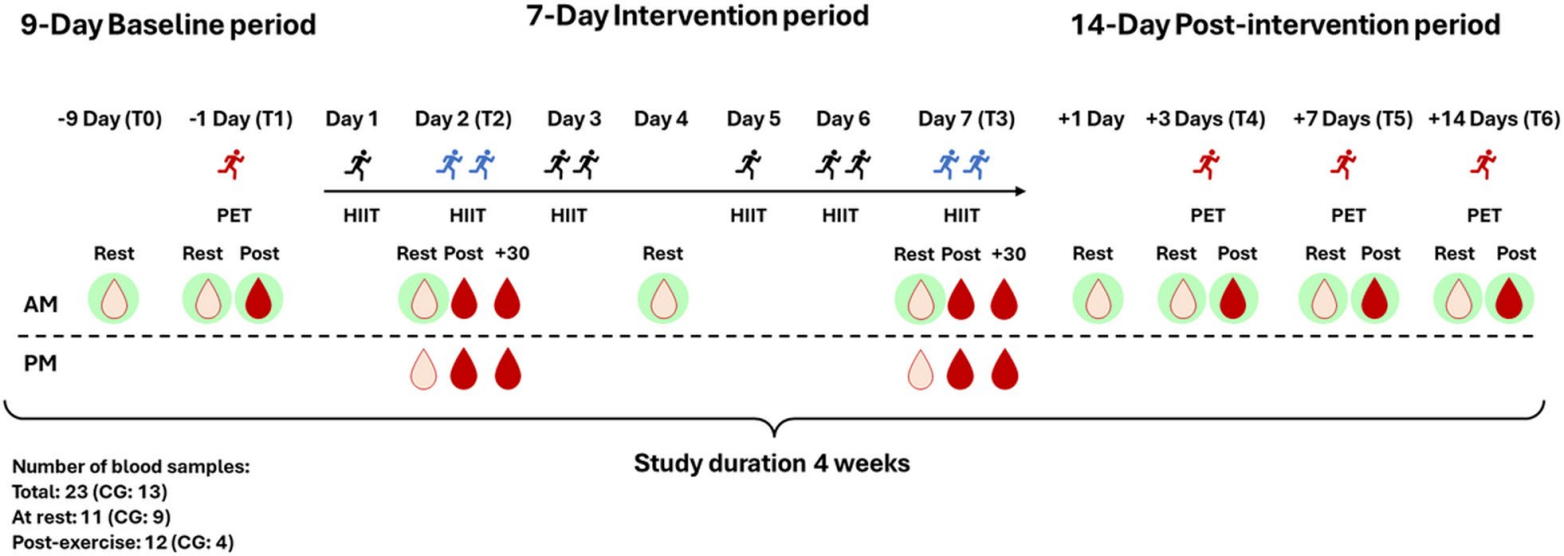
Study design and blood sampling points. High-intensity interval training shock microcycle (HSM) and HSM with additional low-intensity training (HSM + LIT) group each had 23 samples collected, 11 at rest and 12 after PET/HIIT, while the control group (CG) had 13 samples, with 9 at rest and 4 after PET. Blood drops indicate venous blood sampling. The HSM and HSM + LIT include all samples, while the CG group includes only those with a green background. AM samples were taken in the morning, and PM samples in the afternoon/evening. Light red blood drops indicate samples taken at rest, while dark red drops represent samples taken immediately (Post) and 30 min (+ 30) after PET/HIIT. Resting state (R), Physiological exercise testing (PET), and high-intensity interval training (HIIT). The red runner icon stands for PET, the dark runner icon for self-directed HIIT sessions, and the blue runner icon for supervised HIIT sessions. The icon with one dark runner stands for HIIT completed in the morning, while the icon with two runners stands for the first HIIT in the morning and the second in the afternoon

HIIT sessions on T2 and T3 took place on a laboratory treadmill (Saturn, HP Cosmos, Traunstein, Germany). Sessions were scheduled in the morning (between 6 and 10 AM) and in the afternoon/evening (between 3 and 7 PM), with a minimum of 5 h between sessions. During the HIIT sessions, interval intensity was regulated using continuous HR monitoring, while the intensity during active recovery and LIT was controlled based on running velocity corresponding to a lactate threshold of 1.5 mmol/l. Throughout the intervention, participants in both intervention groups were instructed not to undertake any additional training sessions beyond the prescribed HIIT and LIT. Figure 2 outlines the 4-week study design including all blood sampling points.

Venous blood samples were collected at rest at time points T0, T1, T2 (AM and PM), day 4 of the intervention, T3 (AM and PM), day 1 after the intervention, T4, T5, and T6. At time points T1, T4, T5, and T6, blood was also collected immediately post-PET. In addition, blood samples were collected immediately, as well as 30 min after HIIT sessions at time points T2 and T3 (AM and PM). Note that the 30-min post-exercise concentration was measured in the HSM group following a 30-min resting period. In HSM + LIT, the post-30 min concentration was measured immediately after 30 min of LIT. This resulted in a total of 23 blood samples (i.e., 11 samples at rest, 4 after PET, and 8 after HIIT sessions) for both HSM and HSM + LIT. In contrast, the CG had a total of 13 blood samples collected (i.e., 9 samples at rest, and 4 after PET).

In the context of this study, we define acute changes in cfDNA as the exercise-induced responses observed from rest to post-exercise measurements. These include changes in cfDNA concentrations from rest to immediately post-PET at time points T1, T4, T5, and T6, as well as from rest to immediately post and 30 min post-HIIT sessions at T2 and T3 (AM and PM). In contrast, chronic changes refer to alterations in resting cfDNA concentrations observed over the entire study period, independent of acute exercise responses. This includes resting values measured at baseline (T0 and T1), during the intervention period (T2, day 4, and T3, day + 1), and during the post-intervention period (T4, T5, and T6).

### Real time qPCR of cfDNA Concentrations in Venous Plasma

#### Blood Collection and Processing

Venous blood (~ 7.5 ml) was collected from the antecubital vein using S-Monovettes^®^ 7.5 ml K3 EDTA tubes (Sarstedt, Nümbrecht, Germany) and centrifuged at 1600 × g for 10 min at 4 °C. Plasma was carefully aspirated, maintaining a safety margin of at least 2 cm (~ 2.66 ml) above the buffy coat to avoid leukocyte contamination. Subsequently, 150 µl aliquots were transferred into 1.5 ml tubes and stored at − 20 °C according to a validated protocol [31]. The INTEGRA Assist Plus robot was used for the pipetting process [32].

#### Cell-Free DNA Measurement

The cfDNA measurement in this study followed the assay described and validated by Neuberger et al. (2021) [31]. The quantitative polymerase chain reaction (qPCR) reaction mix (5 μl total volume) contained 1 µl diluted plasma, 3.9 µl MasterMix (HiFi buffer, dNTP, SYBR Green Polymerase), and 0.1 µl PrimerMix (forward and reverse primers). The final concentrations included: 1.2 × HiFi buffer (BioCat, Heidelberg, Germany), 0.3 mM dNTP (Carl Roth, Karlsruhe, Germany), 0.15 × SYBR Green (Sigma-Aldrich, Taufkirchen, Germany), 0.04 IU Velocity Polymerase (BioCat, Heidelberg, Germany), and 140 nM of each primer. Primers targeted the hominoid-specific LINE-1 family 2 (L1PA2) elements, amplifying cfDNA fragments of 90 and 222 base pairs. A shared forward primer (5’-TGC CGC AAT AAA CAT ACG TG-3’) was paired with either a 90 bp reverse primer (5’-GAC CCA GCC ATC CCA TTA C-3’) or a 222 bp reverse primer (5’-AAC AAC AGG TGC TGG AGA GG-3’).

The coefficients of variation for both 90 and 222 bp cfDNA assays ranged from 4% to 7% (inter‑assay) and from 3% to 6% (intra‑assay), demonstrating strong reproducibility [31]. These values fall well within accepted reliability thresholds for qPCR methods [33].

#### qPCR Protocol

To ensure reproducibility, each sample was measured in technical duplicates (5 µl per well), alongside negative control samples (H₂O and mouse plasma) and eight reference samples to control for inter-plate variability and monitor contamination. Analyses were performed using a CFX384 Opus™ Real-Time PCR System (Bio-Rad, Munich, Germany). The thermal profile consisted of initial denaturation at 98 °C for 2 min, followed by 35 cycles of 94 °C for 10 s (denaturation), 64 °C for 10 s (annealing), and 75 °C for 10 s (extension). To ensure comparability with previous assays [14, 31], the ramp rate was set to 1.3 °C/s. The protocol concluded with a melting curve analysis (65–95 °C with 0.5 °C increments for 5 s).

#### Calculation of cfDNA Concentration in Plasma Samples

The copy number per reaction was derived using three independent standard curves. This was divided by 5 to obtain the copy number per µl, multiplied by 75 to account for the total plasma dilution (1:15 for plasma, 1:5 for qPCR), and divided by predicted hits (3416 for 90 bp, 3237 for 222 bp). Genome equivalents were multiplied by 3.23 pg, converting the result to ng/ml. If the standard deviation (SD) of the quantification cycle (Cq) values between technical duplicates exceeded 0.4, the sample was re-diluted and reanalysed up to two times. Samples were classified as non-measurable if the Cq SD remained above this threshold.

The cfDNA integrity index was calculated as the ratio of cfDNA concentration from the 222 bp to the 90 bp fragments.

### Statistical Analysis

For data storage, transformation, and analysis, Microsoft Excel (Version 2408, Microsoft Corporation, Redmond, WA) and R software (R Version 4.2.2; RStudio Version 2024.04.2, Inc., Boston, USA) were used. cfDNA concentrations were log-10 transformed, tested for normality using the Shapiro–Wilk test, and visually assessed with Q-Q plots. Parametric tests were used for normally distributed data; otherwise, non-parametric tests were used. Homogeneity of variance was evaluated with the Levene test. The Results section presents the differences in mean cfDNA concentration and integrity index, aggregated by group, test point, measurement point, time of day, and sex.

To evaluate the influence of fixed factors on cfDNA concentrations, multivariate analyses were performed using linear mixed models (lmm) from the lme4 package (version 1.1–35.5), fitted by Restricted Maximum Likelihood [34]. This statistical approach was chosen over repeated-measures ANOVA due to its superior handling of unbalanced data and missing values, and its ability to model both fixed effects (e.g., group, time, measurement point) and random effects (e.g., subject-specific variability), making it more suitable for longitudinal exercise studies with repeated measures. P-values were computed using the Satterthwaite approximation from the lmerTest package (version 3.1-3). Model performance was assessed using the performance package (v0.12.3), including the marginal and conditional R² to quantify explained variance, the root mean square error (RMSE) to evaluate prediction accuracy, and the intra-class correlation coefficient (ICC) to determine group-level consistency. These metrics were used to evaluate the reliability and generalizability of the models.

For acute changes in cfDNA during PET, cfDNA^90^ and cfDNA^222^ served as the dependent variables, respectively. Group (i.e., HSM, HSM + LIT, CG), time point (i.e., T1, T4, T5 and T6), measurement point (i.e., pre and post), and the interaction between group and measurement point were included as fixed effects. For acute changes in cfDNA during HIIT, cfDNA^90^ and cfDNA^222^ concentrations served as dependent variables. Group (i.e., HSM, HSM + LIT, CG), time point (i.e., T2 and T3), time of day (i.e., AM and PM), measurement point (pre, post, and post 30), and the interaction between group and measurement point were included as fixed effects. For chronic cfDNA changes, resting cfDNA^90^ and cfDNA^222^ concentrations served as the dependent variables. Group (i.e., HSM, HSM + LIT, CG), study period (i.e., baseline (T0, T1), intervention (T2, day 4, T3, day + 1) and post intervention period (T4, T5, T6)), time of day (AM and PM), and the interaction between group and study period were included as fixed effects. Subject was included as a random effect in all models.

To assess differences between sexes in cfDNA concentrations, unpaired t-tests were performed. Wilcoxon test was used to analyze differences in TRIMP between HIIT and PET and the acute changes of cfDNA integrity index during PET and HIIT. Kruskal-Wallis test was used to analyze the chronic changes of resting cfDNA integrity index over the study periods. Pairwise correlation analyses between physiological exercise variables measured during (TRIMP, HR_max_, RQ_max_, VO_₂max_, LT and PPO), and immediately after PET (La_peak_, cfDNA^90^, and cfDNA^222^), pooled across all three study groups at time points T1, T4, T5, and T6 were performed using the Spearman rank test.

Alpha level of significance was set to *p* < 0.05 for all statistical analyses and the results are presented as mean ± SD.

## Results

### Participant Characteristics

Out of 35 participants, two participants discontinued and were considered dropouts and two participants had missing blood samples. Thus, 31 (7 women and 24 men) participants were analyzed, while dropouts are described separately (refer to the ‘Dropout Analysis’ subsection in the ‘Results’ section). All participants regularly engaged in endurance activities such as running, trail running, triathlon, canoeing, biking, and football (soccer). Anthropometric characteristics of the participants, including data from the entry PET at T1, are presented in Table 1.

**Table 1:** Participant characteristics and physiological exercise variables at baseline assessment

	Total	HSM	HSM + LIT	CG
Sample size	31	10	10	11
Female (%)	7 (23%)	4 (40%)	1 (10%)	2 (18%)
Age (years)	29 (6)	29 (5)	29 (6)	30 (7)
Height (cm)	177 (8)	173 (8)	179 (7)	180 (7.5)
Weight (kg)	69.4 (8.9)	65.7 (8.0)	71.4 (8.1)	71.6 (9.8)
BMI (kg/m^2^)	22.1 (2.1)	21.6 (2.3)	22.3 (2.1)	22.3 (2.0)
VO_2max_ (ml/min/kg)	60.0 (4.9)	60.3 (6.9)	60.3 (5.2)	59.4 (2.5)
RPE (6–20)	19 (1)	20 (1)	19 (1)	19 (1)
RQ_max_	1.17 (0.05)	1.16 (0.05)	1.18 (0.06)	1.16 (0.05)
HR_max_ (bpm)	190 (8)	189 (6)	191 (11)	189 (8)
LT (km/h)	12.4 (1.4)	12.3 (1.9)	12.6 (1.4)	12.5 (1.0)
PPO (watt/kg)	5.1 (0.5)	5.0 (0.6)	5.2 (0.4)	5.0 (0.4)
La_peak_ (mmol/L)	9.8 (2.4)	9.5 (2.2)	10.7 (2.2)	9.1 (2.7)

Data of the study participants at the baseline assessment (T1). The data are presented as mean (SD) or absolute (N) and relative frequency (%). High-intensity interval training shock microcycle (HSM), HSM with additional low-intensity training (HSM + LIT), and control group (CG). Body mass index (BMI), Maximum oxygen uptake rate (VO_₂max_), Rating of perceived exertion (RPE), Maximum respiratory quotient (RQ_max_), Maximum heart rate (HR_max_), Running velocity at lactate threshold (LT), Peak power output (PPO), and Peak capillary blood lactate concentration (La_peak_)

### Overview of cfDNA Measurements

Out of a total of 603 venous blood samples, 596 (98.8%) were successfully measured using the L1PA2 90 bp, while the 222 bp assays had a similarly high measurability rate with 578 samples (95.9%). A total of 32 blood samples (7 for the 90 bp assay and 25 for the 222 bp assay) remained unmeasurable despite two attempts. Reasons for these failures include contamination of sample materials (e.g., hemolysis and environmental contaminants), and insufficient sample volume. The cfDNA integrity index over all measured samples was 0.39 ± 0.22. A detailed overview of all measured samples, including acute and chronic changes in venous plasma cfDNA (ng/ml) concentrations for each group, is available in the additional files (Supplementary Material).

### Acute Changes in cfDNA Concentrations during Physiological Exercise Testing

The lmm for cfDNA^90^ demonstrated strong performance, explaining 93.5% of the variance with a low prediction error (RMSE = 0.124) and moderate group consistency (ICC = 0.551). Similarly, the lmm for cfDNA^222^ performed well, explaining 89.1% of the variance with a slightly higher RMSE of 0.174 and lower group consistency (ICC = 0.367).

**Fig. 3:**
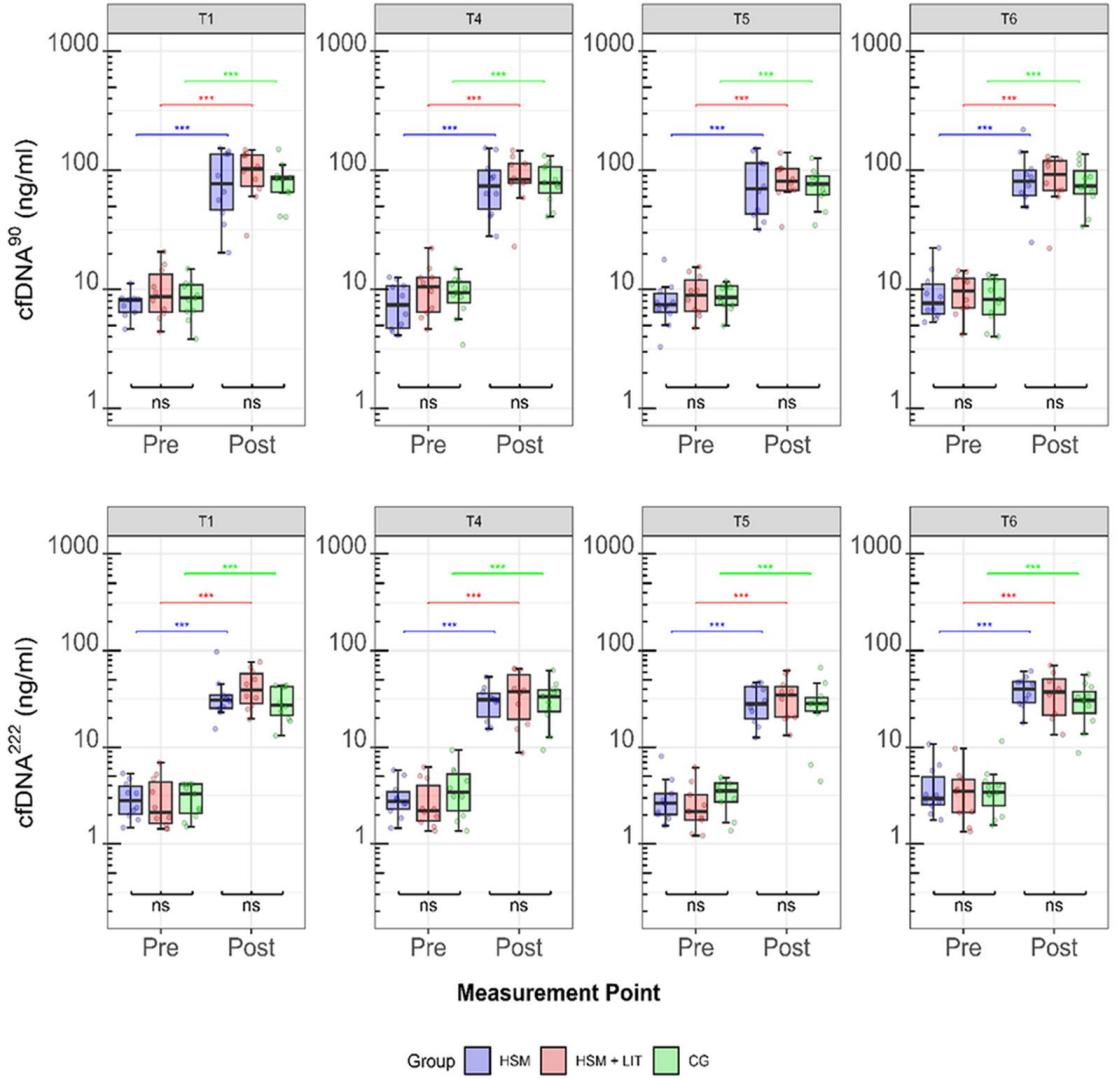
Acute cfDNA Concentration changes during physiological exercise testing (PET). Acute changes in cfDNA concentrations (ng/ml) during PET are shown for both L1PA2 90 (cfDNA^90^) and 222 (cfDNA^222^) base pair assays. Values are presented as mean ± SD. Bonferroni–Holm adjusted p-values: ns ≥ 0.05 and ***p < 0.001. The high-intensity interval training shock microcycle (HSM) is shown in blue, HSM with additional low-intensity training (HSM + LIT) in red, and control group (CG) in green. The time points analyzed were T1, T4, T5, and T6, with two measurement points: pre- and immediately post-PET

Significant effects of the measurement point on cfDNA^90^ (F(1, 209) = 3184, *p* < 0.001) and cfDNA^222^ (F(1, 201) = 1882, *p* < 0.001) were observed reflecting significant increases in overall cfDNA concentrations from pre- to post-PET. Independent of groups and time points, cfDNA^90^ (9.1 ± 3.8 to 86.0 ± 37.4 ng/ml, FC: 10.4-fold) as well as cfDNA^222^ (3.4 ± 2.0 to 34.3 ± 16.5 ng/ml, FC: 12.4-fold) increased from pre- to post-PET (cfDNA^90^: t = 5.0, *p* < 0.001; cfDNA^222^: t = 4.3, *p* = 0.002). No significant effects were found for group on cfDNA^90^ (F(2, 27) = 0.400, *p* = 0.674) and cfDNA^222^ (F(2, 227) = 0.079, *p* = 0.925). In addition, no effects were found for time point on cfDNA^90^ (F(3, 209) = 0.306, *p* = 0.821) and cfDNA^222^ (F(3, 202) = 2, *p* = 0.085). No significant interaction was observed between group and measurement point on cfDNA^90^ (F(2, 209) = 0.136, *p* = 0.873) and cfDNA^222^ (F(2, 210) = 0.142, *p* = 0.868). Post-hoc analyses on the effects of measurement point and group on cfDNA concentrations are illustrated in Fig. 3.

Independent of groups and time points, no significant sex differences were found for resting cfDNA^90^ (males: 9.3 ± 4.1, females: 8.5 ± 2.6 ng/ml; W = 1234, *p* = 0.564) and cfDNA^222^ (males: 3.4 ± 2.1, females: 3.2 ± 1.6 ng/ml; W = 1201, *p* = 0.860). After PET, cfDNA^90^ (males: 91.6 ± 37.9, FC: 9.9-fold, females: 66.6 ± 28.6 ng/ml, FC: 7.8-fold; W = 818, *p* = 0.003) and cfDNA^222^ (males: 36.5 ± 15.9, FC: 10.7-fold, females: 27.2 ± 16.8 ng/ml, FC: 8.5-fold; W = 816, *p* = 0.006) concentrations were significantly higher in male participants. Independent of groups and time points, no significant differences from pre- to post-exercise were found for cfDNA integrity index (0.39 ± 0.20 to 0.45 ± 0.28, FC: 1.4-fold, W: 6268, *p* = 0.121).

### Acute Changes in cfDNA Concentrations during HIIT Sessions

The lmm for cfDNA^90^ demonstrated strong performance, explaining 95.2% of the variance with a low prediction error (RMSE = 0.119) and high group consistency (ICC = 0.696). The lmm for cfDNA^222^ also performed well, explaining 89.1% of the variance with a slightly higher RMSE of 0.192 and moderate group consistency (ICC = 0.505).

**Fig. 4:**
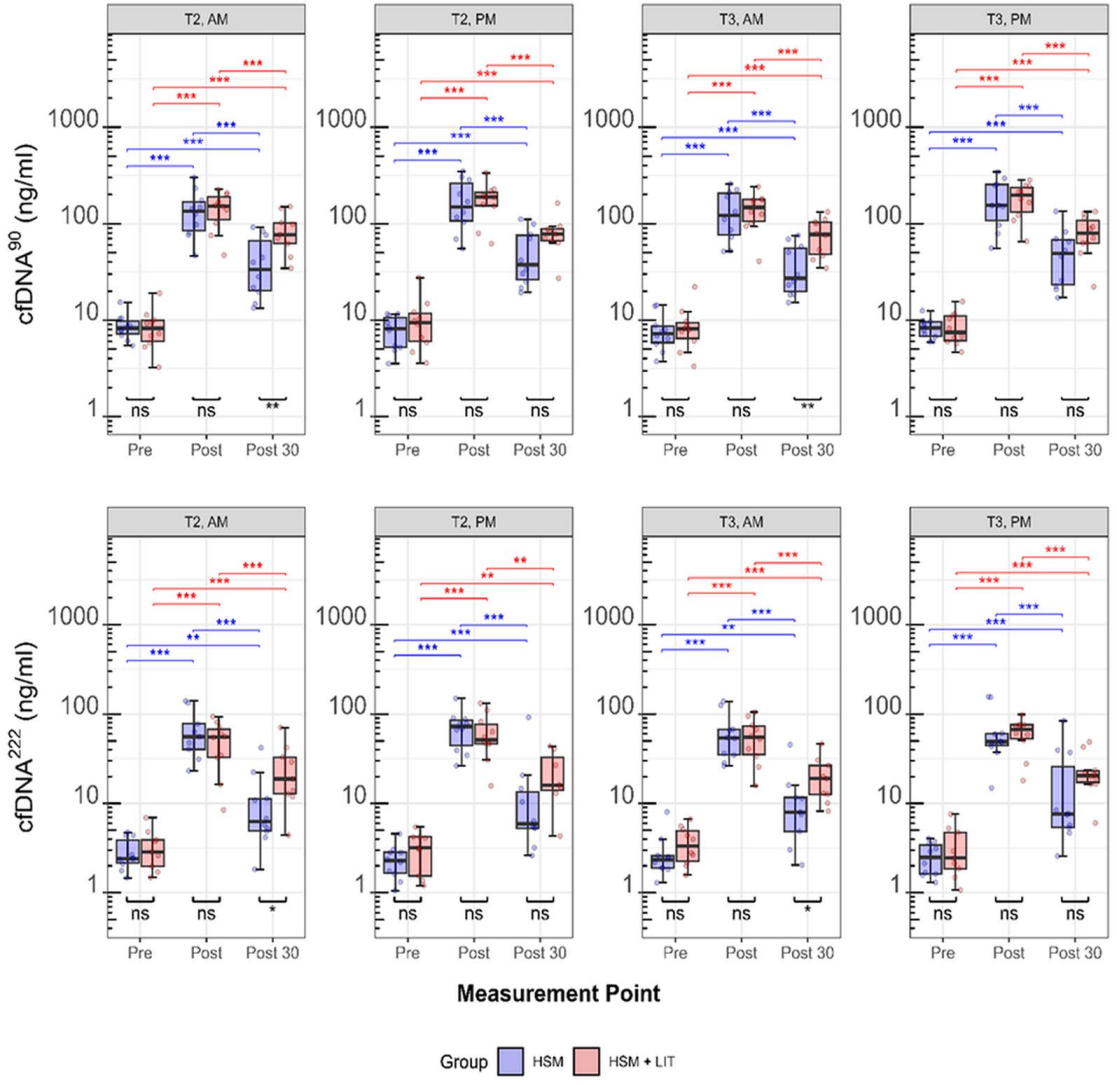
Acute cfDNA Concentration changes during laboratory high-intensity interval training (HIIT) sessions. Acute changes in venous plasma cfDNA concentrations (ng/ml) during laboratory high-intensity interval training (HIIT) sessions are shown for both L1PA2 90 (cfDNA^90^) and 222 (cfDNA^222^) base pair assays. Box plots represent the median, interquartile range (IQR), and whiskers (minimum to maximum range), with individual data points overlaid. Bonferroni-Holm adjusted p-values: ns ≥ 0.05, *p < 0.05, **p < 0.01, ***p < 0.001. The high-intensity interval training Shock Microcycle (HSM) is shown in blue and HSM with additional low-intensity training (HSM + LIT) in red. The time points analyzed were T2 and T3, with three measurement points: pre-, immediately post-, and 30 min post-HIIT. Time of day is indicated as morning (AM) and afternoon (PM)

Significant effects of the measurement point on cfDNA^90^ (F(2, 212) = 2037, *p* < 0.001) and cfDNA^222^ (F(2, 204) = 786, *p* < 0.001) were found. Independent of groups and time points, cfDNA^90^ (8.8 ± 4.1 to 162.3 ± 77.5 ng/ml, FC: 19.8-fold) and cfDNA^222^ (3.0 ± 1.6 to 62.7 ± 34.8 ng/ml, FC: 23.3-fold) increased significantly from pre- to post-HIIT (cfDNA: t = 8.9, *p* < 0.001; cfDNA^222^: t = 4.9, *p* < 0.001). Both cfDNA^90^ (64.1 ng/ml, FC: 7.7-fold) and cfDNA^222^ (62.7 ng/ml, FC: 5.6-fold) remained significantly elevated 30 min post-HIIT compared to rest (cfDNA^90^: t = 15.6, *p* < 0.001; cfDNA^222^: t = 21.2, *p* < 0.001). A statistical trend for time of day on cfDNA^90^ (F(1, 212) = 3, *p* = 0.061) concentrations was found, while no such trend was found for cfDNA^222^ (F(1, 204) = 0.655, *p* = 0.419). Independent of groups and time points, cfDNA^90^ concentrations immediately post-HIIT were higher in PM compared to AM (175.7 ± 83.3 ng/ml, FC: 22.4-fold vs. 148 ± 69.7, FC: 17.2-fold; W = 152, *p* < 0.001). However, 30 min after HIIT, cfDNA^90^ concentrations were no longer statistically different between PM and AM (66.1 ± 37.8 ng/ml, FC: 8.1-fold vs. 62.2 ± 36.7 ng/ml, FC: 7.3-fold; W = 333, *p* = 0.434). No significant effects were found for time point on cfDNA^90^ (F(1, 212) = 0.402, *p* = 0.527) and cfDNA^222^ (F(1, 204) = 0.462, *p* = 0.498). In addition, no effects of group on cfDNA^90^ (F(1, 18) = 1, *p* = 0.199) and cfDNA^222^ (F(1, 18) = 1, *p* = 0.197) were found. A significant interaction effect between measurement point and group was observed for both cfDNA^90^ (F(2, 212) = 28, *p* < 0.001) and cfDNA^222^ (F(2, 204) = 19, *p* < 0.001). cfDNA^90^ concentrations were significantly higher in HSM + LIT compared to HSM 30 min after HIIT (82.1 ± 35.0, FC: 10.0-fold vs. 47.0 ± 30.6, FC: 5.5-fold; W: 194, *p* < 0.001). cfDNA^222^ showed a tendency towards higher concentrations in HSM + LIT compared to HSM 30 min post-HIIT (23.4 ± 14.7, FC: 7.7-fold vs. 20.5 ± 15.2, FC: 5.4-fold; W: 313, *p* = 0.084). Post-hoc analyses on the effects of measurement points and intervention groups on cfDNA concentrations are illustrated in Fig. 4.

Independent of groups and time points, resting cfDNA^90^ (males: 9.1 ± 4.5 ng/ml vs. females: 7.8 ± 2.1 ng/ml; W = 543, *p* = 0.530) and cfDNA^222^ (males: 2.9 ± 1.6 ng/ml vs. females: 3.2 ± 1.4 ng/ml; W = 543, *p* = 0.530) concentrations were not statistically significant different between sexes. cfDNA^90^ (males: 171.1 ± 78.4 ng/ml, FC: 20.1-fold vs. females: 135.9 ± 70.0 ng/ml, FC: 18.8-fold; W = 429, *p* = 0.12) and cfDNA^222^ (males: 64.2 ± 33.1 ng/ml, FC: 24.2-fold vs. females: 58.1 ± 40.0 ng/ml, FC: 20.1-fold; W = 463, *p* = 0.308) concentrations immediately post-HIIT showed no significant sex differences. However, cfDNA^90^ (males: 73.7 ± 36.7 ng/ml, FC: 8.7-fold vs. females 34.5 ± 18.0 ng/ml, FC: 4.6-fold; W = 194, *p* < 0.001) and cfDNA^222^ (20.7 ± 19.2 ng/ml, FC: 7.2-fold) vs. females (13.7 ± 14.4 ng/ml, FC: 4.1-fold; W = 313, *p* = 0.084) were higher in males 30 min post-HIIT.

Independent of groups and time points, cfDNA integrity index did not change from rest to immediately post-HIIT (0.38 ± 0.20 vs. 0.40 ± 0.18; W: 2718, *p* = 0.242). However, significant differences were found 30 min post-HIIT compared to rest (0.38 ± 0.20 vs. 0.30 ± 0.24; W = 3804, *p* < 0.001) and between immediately post-HIIT and 30 min post-HIIT (0.40 ± 0.18 vs. 0.30 ± 0.24; W = 4187, *p* < 0.001).

### Differences in Acute Changes in cfDNA Concentrations Between Exercise Protocols

**Fig. 5:**
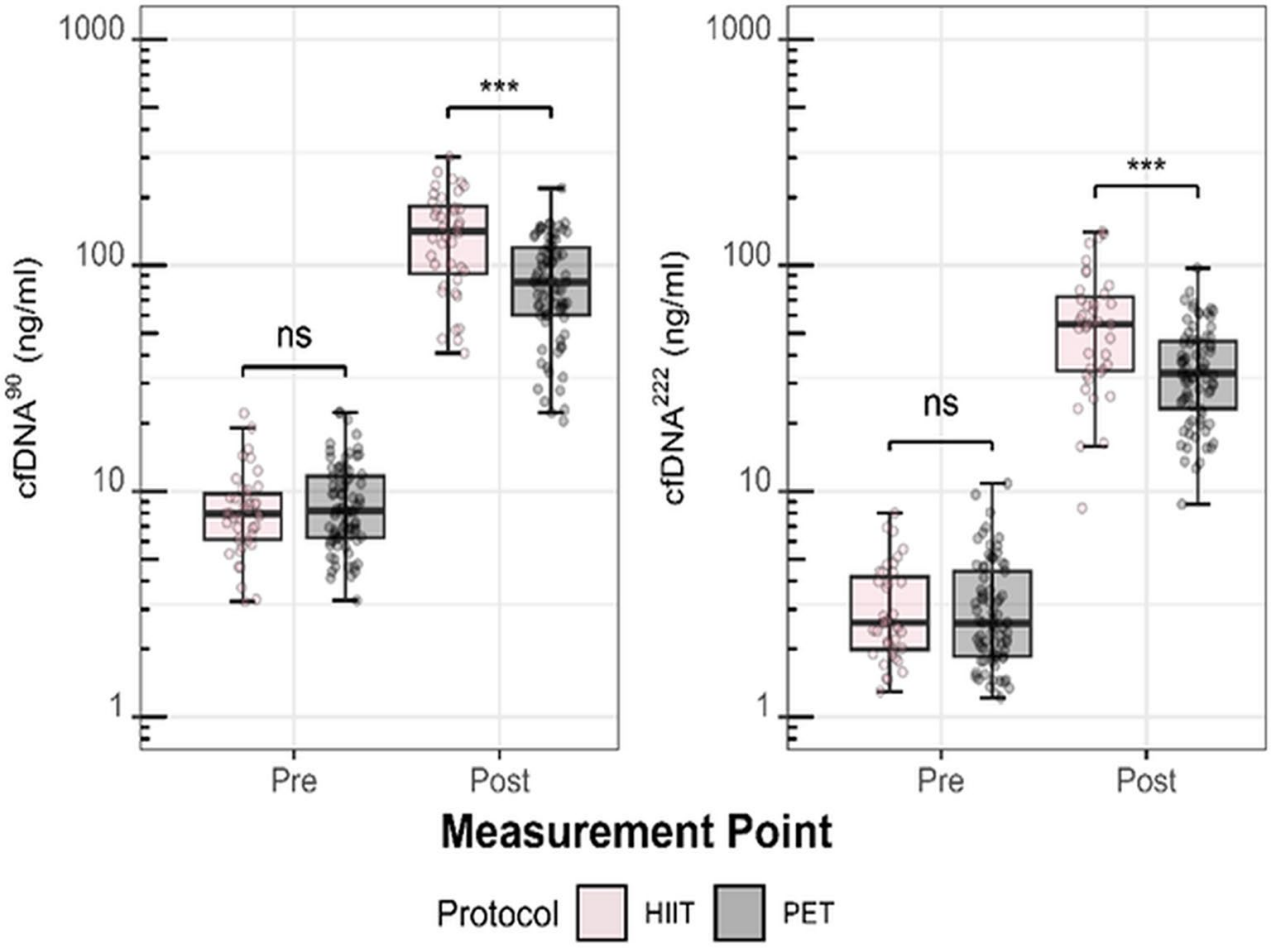
Mean differences in cfDNA Concentrations between high-intensity interval training (HIIT) and physiological exercise testing (PET). Differences in cfDNA concentrations (ng/ml) between HIIT (pink) and PET (grey) are shown for two measurement points: pre- and immediately post-HIIT/PET, using both L1PA2 90 (cfDNA^90^) and 222 (cfDNA^222^) base pair assays. Box plots represent the median, interquartile range (IQR), and whiskers (minimum to maximum range), with individual data points overlaid. Mean comparison analysis was performed using unpaired t-tests. p-values: ns ≥ 0.05, *p < 0.05, **p < 0.01, ***p < 0.001

Figure 5 presents pre- and post-exercise concentrations from both intervention groups for PET (T1, T4, T5, and T6) and HIIT (T2 and T3). Only data from AM HIIT sessions were analyzed to control for potential time of day effects, resulting in 40 cfDNA observations from HIIT sessions compared to 80 observations from PET sessions.

Independent of groups and time points, cfDNA^90^ concentrations were significantly higher after HIIT compared to PET (148.8 ± 69.7 ng/ml, FC: 17.1 vs. 88.8 ± 41.2 ng/ml, FC: 10.4; t: 4, *p* < 0.001). Similarly, cfDNA^222^ concentrations were significantly higher after HIIT compared to PET (59.4 ± 34.1 ng/ml, FC: 20.2 vs. 36.4 ± 17.4 ng/ml, FC:12.4; t: 3, *p* < 0.001). TRIMP was significantly higher during HIIT (78.1 ± 9.7) compared to PET (52.9 ± 7.4, w: 3200, *p* < 0.001).

### Chronic cfDNA Changes

The lmm for cfDNA^90^ showed moderate performance, explaining 56.2% of the variance with a prediction error of RMSE = 0.107 and moderate group consistency (ICC = 0.553). The lmm for cfDNA^222^ showed lower performance, explaining 42.3% of the variance with a higher RMSE of 0.167 and lower group consistency (ICC = 0.395).

**Fig. 6:**
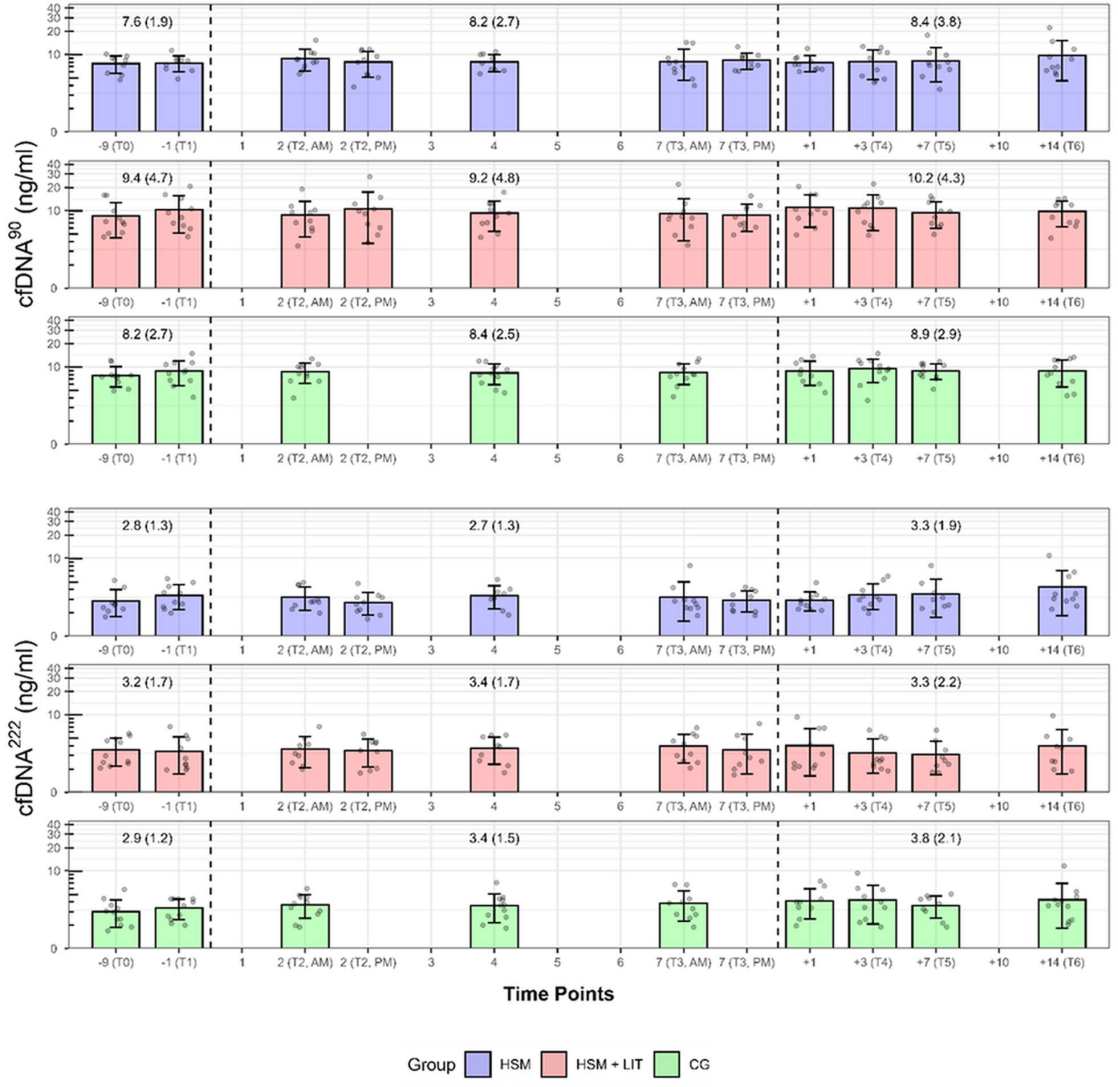
Chronic changes in resting cfDNA Concentrations. Chronic changes in resting venous plasma cell-free DNA concentrations (ng/ml) over the study course are presented for both L1PA2 90 (cfDNA^90^) and 222 (cfDNA^222^) base pair assays. Values are presented as mean ± SD. Furthermore, cfDNA concentrations (ng/ml) are provided for the baseline, intervention, and post-intervention periods. High-intensity interval training shock microcycle (HSM) shown in blue, high-intensity interval training shock microcycle with additional low-intensity training (HSM + LIT) shown in red and control group (CG) in green. The time points include: -9 Day (T0), -1 Day (T1), 2 Day (T2), 7 Day (T3), + 1 Day, + 3 Day (T4), + 9 Day (T5), + 10 Day, and + 14 Day (T6)

No significant effects were found for group (F(2, 28) = 0.424, *p* = 0.659), study period (F(2, 276) = 1.271, *p* = 0.282), or time of day (F(1, 276) = 1.136, *p* = 0.288) on resting cfDNA^90^ concentrations. In addition, no significant interaction effects between group and study period (F(4, 276) = 0.236, *p* = 0.918) were found. Similarly, for cfDNA^222^, no significant effects for group (F(2, 32) = 0.516, *p* = 0.602), study period (F(2, 266) = 2.485, *p* = 0.085), or time of day (F(1, 266) = 0.787, *p* = 0.376) were found. No significant interactions were observed between group and study period (F(4, 266) = 1.641, *p* = 0.164). cfDNA trajectories at rest over the study course are shown in Fig. 6.

cfDNA integrity index at rest remained stable across the study periods, with values of 0.36 at baseline, 0.38 during the intervention period, and 0.40 post-intervention (H = 1.263, *p* = 0.532).

### Relationship between cfDNA Concentrations and Established Physiological Exercise Variables

**Fig. 7:**
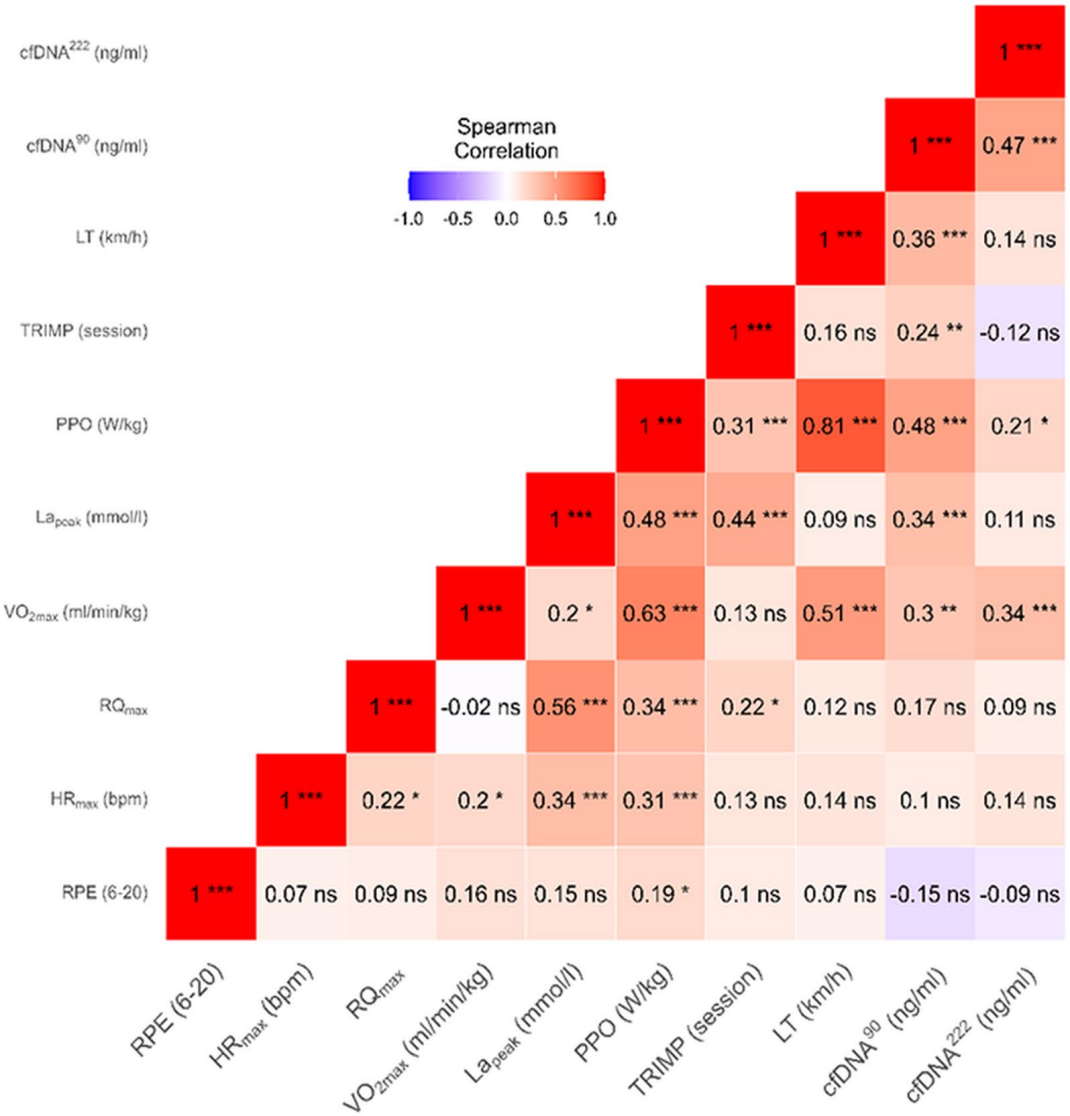
Pairwise correlation analysis between cfDNA Concentrations and physiological exercise variables. A pairwise correlation analysis was conducted using the Spearman rank method to examine relationships between established physiological exercise variables and circulating cell-free DNA (cfDNA) concentrations (ng/ml), measured in venous plasma immediately post-PET during all physiological exercise tests at T1, T4, T5, and T6. The 90 (cfDNA^90^) and 222 (cfDNA^222^) base pair assays were used. All p-values were Bonferroni–Holm adjusted for 45 comparisons. Adjusted p-values are reported as follows: ns ≥ 0.05, *p < 0.05, **p < 0.01, ***p < 0.001. Rating of perceived exertion (RPE), maximum heart rate (HR_max_), maximum respiratory quotient (RQ_max_), maximum oxygen uptake rate (VO_₂max_), peak capillary blood lactate concentration (La_peak_), peak power output (PPO), training impulses during PET (TRIMP), and running velocity at lactate threshold (LT)

Correlation analysis was performed to examine the relationship between established physiological exercise variables: RPE, HR_max_, RQ_max_, VO₂_max_, La_peak_, PPO, TRIMP, LT and post-PET cfDNA concentrations across four time points T1, T4, T5 and T6, totaling 45 comparisons (Fig. 7).

cfDNA^90^ showed a moderate correlation with PPO (*r* = 0.48, *p* < 0.001), LT (*r* = 0.36, *p* < 0.001), La_peak_ (*r* = 0.34, *p* < 0.001), VO_₂max_ (*r* = 0.30, *p* < 0.01), and a low correlation with TRIMP (*r* = 0.24, *p* < 0.01). cfDNA^222^ showed a moderate correlation with cfDNA^90^ (*r* = 0.47, *p* < 0.001) and VO_₂max_ (*r* = 0.34, *p* = 0.001), as well as a low correlation with PPO (*r* = 0.21, *p* = 0.05).

### Dropout Analysis

Two participants who discontinued the study on days 3 and 4 of the intervention period were analyzed separately in this section. One participant (P1) withdrew due to iliotibial band syndrome, and the second participant (P2) discontinued due to nausea and headache (both HSM + LIT). Both P1 and P2 showed regular cfDNA^90^ concentrations between 6.9 and 7.4 ng/ml (P1) and between 5.2 and 10.7 ng/ml (P2) at rest. Similarly, resting cfDNA^222^ concentrations ranged from 2.8 to 3.8 ng/ml (P1) and from 2.3 to 3.3 ng/ml (P2). Interestingly, P1 showed exceptionally high cfDNA^90^ concentrations of 262.6 ng/ml (FC: 37.9-fold) after T2 AM HIIT and 390.0 ng/ml (FC: 55.7-fold) after T2 PM HIIT. In line with these findings, cfDNA^222^ concentrations in P1 increased to 106.2 ng/ml (FC: 35.4-fold) at T2 AM and to 209.1 ng/ml (FC: 72.1-fold) at T2 PM. In contrast, post-exercise concentrations for P2 were comparable to those found in the overall results (cfDNA^90^ FC between 5.1-fold and 16.4-fold, cfDNA^222^ FC between 4.9-fold and 16.9-fold).

## Discussion

### Principal Findings

This is the first study that examines both acute and chronic changes in cfDNA in trained athletes during a 7-day HIIT shock microcycle with or without additional LIT. We observed acute marked increases in venous cfDNA concentrations following both PET and HIIT. Acute cfDNA increases were dependent on the exercise protocol, with higher increases during HIIT and, particularly, during afternoon sessions. In addition, we found that males exhibited higher cfDNA increases compared to females following exercise. Notably, no chronic cfDNA increases were observed throughout the study period, regardless of the study group. Significant correlations were identified between cfDNA concentrations and physiological exercise variables such as PPO, LT, and VO_₂max_ during PET. Interestingly, one participant who discontinued the study due to a muscle injury showed exceptionally high cfDNA concentrations, suggesting a possible link between elevated cfDNA concentrations and signs of overload.

### Acute Changes in cfDNA

Our findings of acute cfDNA increases after both PET and HIIT align with previous findings [14, 22] suggesting cfDNA as a highly sensitive marker for acute exercise load. Beiter et al. [35] reported a 9.9-fold increase in cfDNA after exhaustive short-term treadmill exercise, which is roughly consistent with the 10.4 -fold increase in our study. These increases are higher compared to other exercise modalities such as resistance exercise (FC: 3.3-fold [23]), all-out cycling (FC: 4.4 -fold [36]), or repeated drop jumps (FC: 5-fold [37]), but considerably lower than those reported following a football game (FC: 23-fold [22]) or a marathon run (FC: 39-fold [17]). Acute cfDNA increases and a subsequent decline observed in our study suggest that the primary mechanism for exercise-induced cfDNA release is likely due to the active secretion by NETs [38]. Recent studies determined that exercise mainly induces a release of DNA from neutrophils, whereby vital and non-vital NETosis could be a major pathway [18, 39]. NETs are a crucial component of the innate immune response, where neutrophils release their DNA to form an antimicrobial mesh that captures and neutralizes invading microorganisms [38]. Notably, cfDNA is free circulating and only a minor amount is associated with large or small extracellular vesicles [40, 41].

While the previous evidence [17, 38, 39] and our findings suggest that NETs are a likely source of cfDNA following acute exercise, we acknowledge that other physiological mechanisms may also contribute to cfDNA release [42]. These include apoptosis as part of normal tissue turnover [43], active secretion from viable immune or endothelial cells under stress [18], and possibly low levels of mitochondrial or cardiac cfDNA, particularly in response to oxidative stress [44]. Although these pathways differ in fragmentation patterns and kinetics, they may overlap under high-intensity conditions. Nevertheless, the observed overall mean cfDNA integrity index of 0.39 supports a predominance of short fragments, consistent with NET-associated or active release mechanisms, rather than necrosis or pathological cell lysis [14, 36].

Previous studies that investigated the effects of exhaustive incremental exercise testing on cfDNA [14, 25] revealed strong correlations with e.g., La, HR, VO_2max_, or total energy expenditure, suggesting that exercise intensity significantly affects cfDNA increases. Haller et al. [25] observed in a controlled test-retest setting of moderate aerobic running that both intensity and duration contribute to cfDNA increases. Here, we observed significantly higher cfDNA responses after HIIT compared to PET. Since HIIT also showed higher TRIMP values, this supports the assumption that exercise intensity is a significant factor contributing to the increases in cfDNA. Interestingly, cfDNA declined despite 30 min of low-intensity running in the HSM + LIT group. This finding is new as previous studies consistently reported increases of cfDNA even during moderate exercise [25]. cfDNA concentrations were higher in HSM + LIT compared to HSM after 30 min of LIT compared to passive rest. Beiter et al. [35] reported a rapid clearance of cfDNA from plasma after only 30 min of passive recovery following exhausting short-duration treadmill exercise. It is well known that physical exercise stimulates DNase activity, which counteracts cfDNA increases [39, 45]. Enhanced DNase activity has been observed both immediately post-exercise and up to 30 min post-exercise, leading to rapid cfDNA degradation [46]. In this respect, 30 min of moderate running appears to still stimulate higher cfDNA concentrations compared to passive rest. However, DNase activity reduces cfDNA concentrations below peak cfDNA concentrations observed immediately after HIIT. Observations of rapid increases with a subsequent less pronounced increase due to constant exercise were previously reported [25], hinting also at a time-delayed increase in DNase activity. The rate of degradation in an exercise setting thus appears to depend largely on how much cfDNA has already been released.

Another novel finding from our study are the higher increases in cfDNA^90^ during afternoon (FC: 22.4-fold) compared to morning HIIT sessions (FC: 17.2-fold). cfDNA concentrations are governed by the dynamic balance between its release from cells and its clearance from the bloodstream via DNase activity, liver and to a lesser extent by the kidneys and urine [46, 47]. Breitbach et al. [12] propose a multifactorial model in which metabolic-, oxidative-, mechanic-, hormonal-, and thermal stress caused by acute physical exhaustion, trigger an aseptic inflammatory response that leads to an increase in cfDNA from immune-competent cells. Previous studies have highlighted the relationship between cfDNA concentrations and endogenous pyrogens [48, 49], reactive oxygen species [50, 51] and pro-inflammatory cytokines [52].

Therefore, the repeated training session on days 2 and 7 of the intervention period may have led to increased metabolic and oxidative stress in the afternoon [53, 54]. Thus far, only a few studies have examined the effects of multiple training sessions within a single day [55–57]. These studies suggest that repeated exercise can enhance immune responses and stress hormone release, which could explain the higher post-exercise cfDNA concentrations in the afternoon. Importantly, while we observed higher post-exercise cfDNA concentrations in the afternoon, resting cfDNA concentrations did not differ between morning and afternoon sessions, for either the 90 bp or the 222 bp fragments. This was consistent across both intervention groups (HSM and HSM + LIT) and supports current evidence suggesting no consistent circadian pattern in cfDNA concentrations [58].

Sex differences found in the present study were previously reported by Blumkaitis et al. [59] and align with a recent study reporting greater exercise-induced increases in cfDNA concentrations in men compared to women [60]. These differences may be attributed to the longer test duration during PET and a greater absolute PPO during PET and HIIT in male participants. Hormonal variations, including fluctuations in the menstrual cycle [61] could potentially contribute to disparities in relative changes in cfDNA between the sexes, as estrogen modulates immune and inflammatory activity [61]. Specifically, estrogen has been shown to reduce neutrophil activation and NET formation, potentially attenuating cfDNA release during exercise [62]. However, the current literature on this topic remains limited and warrants further investigation [58]. In our study, participants voluntarily log their menstrual cycles (onset and end of menstruation), but exercise and performance assessments were not adjusted for the phases of the menstrual cycle. Therefore, the data do not allow to say whether menstrual cycle or use of hormonal contraceptives affects cfDNA kinetics.

Typically, cfDNA integrity increases after physical exercise, reflecting tissue damage or cell death due to the physiological stress of high-intensity exercise [14, 36]. Exhaustive exercise has been associated with leukocyte activation and inflammation [63], oxidative stress [64], and both mechanical and metabolic cell damage [65]. These factors likely contribute to processes equal to pathological conditions thereby shifting the cfDNA size spectrum to longer fragments until DNase I converts this effect during recovery [63–65]. Interestingly, recent findings by Stawski et al. (2019) challenge this traditional view by showing a decrease in cfDNA integrity following exercise, which was negatively associated with oxidative stress and phagocyte activation [19]. This suggests that cfDNA integrity is not only influenced by the extent of cellular damage but also by post-release enzymatic degradation dynamics and immune activation. In our study, we observed no statistically significant increase in cfDNA integrity following either the PET or HIIT protocols. However, 30 min post-HIIT, cfDNA integrity index decreased below baseline, suggesting a faster clearance of longer compared to shorter cfDNA fragments. The slightly delayed activation of DNase compared to cfDNA release might explain the elevated DNA integrity immediately post-exercise and the subsequent minimum values observed after 30 min of recovery. Taken together, these observations support the interpretation that cfDNA integrity reflects a complex interplay of release mechanisms and clearance processes, and not merely the presence or absence of tissue damage.

The dropout analysis revealed distinct cfDNA responses in P1, who discontinued due to iliotibial band syndrome. Unlike P2, who showed cfDNA concentrations in line with the overall findings, P1 displayed significantly elevated cfDNA concentrations following PET and HIIT sessions. The 29.8-fold and 55.7-fold increases in cfDNA^90^, along with the 21.4-fold and 72.1-fold increases in cfDNA^222^, suggest an abnormal physiological response linked to muscle injury. These findings underscore the potential of cfDNA as a biomarker for exercise-induced muscle injury [37, 66]. However, to date, no studies have documented such acute and pronounced cfDNA elevations associated with muscle injury. Further research is needed to elucidate the underlying mechanisms connecting elevated cfDNA concentrations with exercise-induced muscle damage.

### Chronic Changes in cfDNA

Due to a short half-life time, acute increases in cfDNA usually return to baseline within minutes or several hours post-exercise [12]. Chronically elevated cfDNA concentrations have been proposed to indicate a quasi-pathological state of overtraining, given their association with various diseases and pathological conditions [24]. Those increased cfDNA concentrations at rest are likely the result of apoptotic cell death triggered by leukocytes or lymphocytes; a process that typically takes several hours [22, 38]. We could not identify chronic cfDNA changes in our study, which could be related to the short duration of HIIT sessions, the short study period of 7 days, and the fact that we did not observe any overtraining-related conditions in our study participants. Additionally, the timing of resting blood sampling might have missed transient fluctuations in cfDNA occurring during recovery. Given the limited number of studies investigating chronic cfDNA changes, particularly in endurance training settings, our understanding of chronic cfDNA changes is limited, thus far. Chronic cfDNA increases may occur in response to high metabolic or mechanical stress such as those experienced during periodized resistance training [20, 24] and repeated football trainings or matches [13, 27]. Fatouros et al. [24] reported increased uric acid and cfDNA concentrations following increased training loads in weightlifters over several weeks, which is indicative of an increased production of reactive oxygen species. Similar results were reported by Gentles et al. [27], who observed a chronic increase in cfDNA at rest during a regular football season. Oxidative stress is believed to prompt the infiltration of neutrophils and macrophages into the muscle tissue [67], leading to apoptosis and necrosis in the hours following exercise [12]. In healthy individuals, cfDNA released into circulation binds to surface proteins on cells such as white blood cells. However, when the binding capacity of these cells is exceeded, excess cfDNA remains circulating in the plasma [12]. The absence of significant changes in resting cfDNA concentrations over time or between groups in our study might indicate a lower level of aseptic inflammation in our participants compared to those in the studies by Fatouros et al. [24] or Gentles et al. [27]. This is plausible, as Fatouros et al. [24] applied a deliberately excessive training stimulus over several microcycles across 12 weeks, progressively increasing strength training loads to evaluate whether cfDNA is sensitive enough to detect overtraining. Gentles et al., [27] in turn, observed increased resting cfDNA concentrations during a regular football season, likely due to the higher cumulative work load and extended monitoring period. In contrast, our study investigated a 7-day HSM or regular endurance training, during which no clinical or performance-related signs of overtraining were observed, except for one dropout related to a muscle injury.

### Practical Implications for Training Load Management

The present findings support the potential of cfDNA as a responsive biomarker [68] for acute exercise-induced stress, with acute increases showing significant correlations with PPO, LT, and VO₂_max_. The higher cfDNA concentrations in afternoon compared to morning HIIT sessions may reflect short-term load accumulation. Additionally, the exceptionally high cfDNA concentrations in a participant who withdrew due to muscle injury suggest potential for cfDNA as a diagnostic marker to help identify excessive stress or poor recovery. Notably, no chronic cfDNA changes were observed during or after the HIIT shock microcycle, indicating that short-term high-intensity phases may not induce sustained cfDNA alterations in well-trained athletes. Consistently higher acute cfDNA responses in males compared to females further highlight the relevance for potential sex-specific monitoring. However, these findings should be confirmed in larger cohorts before practical application.

Although cfDNA shows promise as an early indicator of acute physiological responses, its widespread application in sports practice remains limited by methodological challenges [69]. The pre-analytical handling and qPCR-based analysis are time- and resource-intensive, which currently hinders real-time monitoring. Future developments should aim to establish minimally invasive sampling techniques [70] and point-of-care diagnostics [71] to facilitate cfDNA integration into routine athlete monitoring. These advancements may enable individualized training load management strategies, with the long-term goal of optimizing performance while minimizing injury risk [3].

### Limitations

As this study is descriptive in nature, the lack of detailed exercise-physiological and molecular-biological insights emphasizes the need for further research. Future studies should explore the molecular and cellular mechanisms underlying cfDNA release and clearance, including their associations with load-sensitive biomarkers such as IL-6, heat shock proteins, and myeloperoxidase [69], to deepen our understanding of exercise-induced stress. The 7-day intervention period, the focus on trained endurance athletes, and the unbalanced sex distribution (25 men, 8 women) limit the generalizability of the findings. Moreover, the lack of cfDNA measurements 30 min post-PET and the absence of a passive recovery phase followed by cfDNA assessment in the HSM + LIT group (where the 30-minute post-HIIT cfDNA concentration was measured after low-intensity exercise without rest) restricts the evaluation of cfDNA recovery between protocols. Another limitation is the absence of standardized training specifications for the CG throughout the entire study and for the intervention groups during the baseline phase and 14-day post-intervention period. While this reflects participants’ habitual endurance training, it introduces uncertainty regarding individual training volumes and their potential influence on cfDNA concentrations. However, to minimize the influence of exercise on cfDNA concentrations at rest, all participants, including those in the CG, were instructed to refrain from training on the day before each of the physiological exercise tests.

## Conclusions

This is the first longitudinal, standardized running HIIT study to examine acute and chronic changes in cfDNA concentrations. The observed acute changes, coupled with correlations to performance metrics, highlight the potential of cfDNA as a sensitive marker for monitoring acute exercise load. The absence of chronic cfDNA increases indicates that a longer intervention period or higher cumulative loads may be required to induce sustained elevations. Our findings provide preliminary evidence that a cumulative training load within a single day may increase cfDNA release, though the exact physiological mechanisms remain unclear. Sex-specific differences in acute cfDNA responses, likely associated with the higher PPO and longer test durations in males, are consistent with previous findings. Future research should focus on clarifying the underlying mechanisms and investigating the practical applications of these findings within exercise physiology.

## Supplementary Information

Additional file 1.

## Data Availability

The datasets used and/or analyzed during the current study are available from the corresponding author on reasonable request.

## Abbreviations

HIIT: High-intensity interval training
SM: Shock microcycle
HSM: High-intensity interval training shock microcycle
LIT: Low-intensity training
HSM + LIT: High-intensity interval training shock microcycle with additional low-intensity training
CG: Control group
TP: Time point
MP: Measurement point
AM and PM: Time of day (AM = morning, PM = afternoon)
RCT: Randomized controlled trial
HR: Heart rate
HR_max_: Maximum heart rate
PPO: Peak power output
VO_₂max_: Maximum oxygen uptake rate
RQ_max_: Maximum respiratory quotient
La: Capillary blood lactate concentration
La_peak_: Peak capillary blood lactate concentration
LT: running velocity at lactate threshold
TRIMP: Training impulse
cfDNA: Circulating cell-free DNA
cfDNA^90^: Circulating cell-free DNA measured with L1PA2 90 base pair assay
cfDNA^222^: Circulating cell-free DNA measured with L1PA2 222 base pair assay
qPCR: Quantitative real-time polymerase chain reaction
DNase: Deoxyribonuclease
NETs: Neutrophil extracellular traps
RPE: Rating of perceived exertion
BIA: Bioelectrical impedance analysis
BMI: Body mass index
ECG: Electrocardiogram
lmm: Linear mixed models
RMSE: Root mean square error
ICC: Intra-class correlation coefficient
