# Supplementary material for: Acute and Chronic Effects of a High-Intensity Interval Training Shock Microcycle on Cell-Free DNA: A Randomized Controlled Trial
Source: Sports Med Open. 2025 Nov 21;11:132. doi: 10.1186/s40798-025-00923-9 (PMC12638572; doi:10.1186/s40798-025-00923-9)
Supplement: Supplementary file 1 — Additional file 1. [file 40798_2025_923_MOESM1_ESM.pdf]

Supplementary Material

Table 1: Acute and chronic changes in cfDNA^{90} (ng/ml) concentration.

	HSM				HSM+LIT				CG			
**Time Point**	**N**	**cfDNA^{90} (ng/ml)**	**FC^{acute}**	**FC^{chronic}**	**N**	**cfDNA^{90} (ng/ml)**	**FC^{acute}**	**FC^{chronic}**	**N**	**cfDNA^{90} (ng/ml)**	**FC^{acute}**	**FC^{chronic}**
**-9 (T0)**	9	7.5 (2.0)			10	8.5 (4.2)			11	7.7 (2.3)		
**-1 (T1, Pre)**	9	7.7 (1.9)			10	10.3 (5.3)			11	8.7 (3.1)		
**-1 (T1, Post)**	10	88.3 (50.6)	12.9 (6.0)		10	100.5 (39.6)	12.1 (5.8)		11	82.4 (30.9)	10.5 (4.8)	
**2 (T2, AM, Pre)**	10	9.1 (3.0)		1.2 (0.3)	10	8.8 (4.4)		1.0 (0.3)	11	8.6 (2.5)		1.1 (0.3)
**2 (T2, AM, Post)**	10	153.6 (89.5)	16.1 (5.4)		10	146.7 (58.0)	17.5 (4.6)					
**2 (T2, AM, Post 30)**	10	43.5 (29.8)	4.5 (2.4)		10	87.1 (40.5)	10.8 (4.4)					
**2 (T2, PM, Pre)**	10	7.6 (2.8)		1.0 (0.3)	9	10.5 (6.9)		1.2 (0.8)				
**2 (T2, PM, Post)**	10	167.6 (96.4)	22.8 (11.6)		10	180.2 (77.2)	19.8 (9.0)					
**2 (T2, PM, Post 30)**	10	52.8 (33.9)	6.6 (2.7)		10	81.5 (36.1)	9.9 (3.0)					
**4**	9	7.9 (2.0)		1.1 (0.2)	9	9.2 (4.0)		1.1 (0.3)	11	8.3 (2.5)		1.0 (0.4)
**7 (T3, AM, Pre)**	10	8.4 (3.5)		1.1 (0.3)	10	9.4 (5.3)		1.0 (0.2)				
**7 (T3, AM, Post)**	10	149.8 (81.3)	17.8 (7.2)		10	145.2 (55.2)	17.2 (6.4)					
**7 (T3, AM, Post 30)**	10	41.8 (22.4)	5.0 (2.0)		10	78.8 (32.6)	9.3 (2.9)					
**7(T3, PM, Pre)**	10	7.9 (2.2)		1.1 (0.4)	10	8.4 (3.3)		1.0 (0.3)	11	8.4 (2.6)		1.0 (0.2)
**7 (T3, PM, Post)**	10	173.1 (97.7)	22.1 (11.7)		10	182.1 (71.5)	23.9 (12.7)					
**7 (T3, PM, Post 30)**	10	50.1 (37.8)	6.0 (3.4)		9	81.5 (36.3)	8.1 (4.1)					
**+1**	10	7.7 (1.8)		1.1 (0.4)	9	11.0 (5.0)		1.3 (0.4)	9	8.7 (3.1)		1.1 (0.4)
**+3 (T4, Pre)**	10	8.0 (3.4)		1.1 (0.4)	10	10.7 (5.3)		1.2 (0.3)	11	9.4 (3.2)		1.2 (0.4)
**+3 (T4, Post)**	10	82.0 (43.4)	10.1 (6.3)		10	90.8 (36.2)	9.9 (6.3)		11	83.4 (29.9)	10.0 (5.5)	
**+7 (T5, Pre)**	10	8.2 (4.0)		1.1 (0.4)	10	9.4 (3.7)		1.1 (0.3)	11	8.8 (2.1)		1.1 (0.3)
**+7 (T5, Post)**	10	82.5 (45.9)	10.6 (5.0)		8	84.7 (29.2)	9.8 (4.7)		11	76.2 (25.7)	9.0 (3.7)	
**+14 (T6, Pre)**	10	9.7 (5.3)		1.3 (0.6)	10	9.7 (3.6)		1.1 (0.4)	11	8.8 (3.4)		1.1 (0.5)
**+14 (T6, Post)**	10	91.9 (55.2)	10.9 (7.1)		10	89.9 (35.8)	9.7 (3.6)		11	81.1 (32.8)	11.1 (8.5)	

cfDNA^{90}: circulating cell-free DNA concentration (ng/ml) measured by L1PA2 90 base pair assay and the mean acute and chronic fold changes are presented as mean (SD). FC^{acut}, acute fold changes of cfDNA^{90} based on Post/Pre or Post 30/Pre ratio for each time point. FC^{chronic}, chronic fold changes in cfDNA^{90} based on T0 Pre/T1 Pre, T0 Pre/T2 Pre ratio etc. High-intensity interval training shock microcycle (HSM), HSM with additional low-intensity training (HSM+LIT) and Control group (CG). Time point: time points during the study, AM and PM: sessions that took place in the morning (AM) and afternoon (PM), Pre, Post and Post 30: immediately before, after and 30 minutes after the exercise test. N: number of participants.

Table 2. Acute and chronic changes in cfDNA^{222} (ng/ml) concentration.

	HSM				HSM + LIT				CG			
**Time Points**	**N**	**cfDNA^{90} (ng/ml)**	**FC^{acute}**	**FC^{chronic}**	**N**	**cfDNA^{90} (ng/ml)**	**FC^{acute}**	**FC^{chronic}**	**N**	**cfDNA^{90} (ng/ml)**	**FC^{acute}**	**FC^{chronic}**
**-9 (T0)**	9	2.5 (1.2)			10	3.3 (1.5)			11	2.7 (1.3)		
**-1 (T1, Pre)**	10	3.1 (1.3)			10	3.1 (1.9)			11	3.0 (1.1)		
**-1 (T1, Post)**	10	36.3 (22.8)	19.1 (14.4)		10	43.8 (19.5)	18.3 (12.5)		11	29.9 (11.2)	11.4 (5.2)	
**2 (T2, AM, Pre)**	10	2.9 (1.2)		1.3 (0.4)	9	3.3 (1.7)		1.2 (0.7	11	3.4 (1.4)		1.2 (0.7)
**2 (T2, AM, Post)**	9	68.0 (42.2)	24.8 (13.3)		10	50.8 (27.6)	15.0 (9.1)					
**2 (T2, AM, Post 30)**	10	11.5 (12.1)	5.1 (4.7)		9	26.2 (20.3)	8.9 (7.6)					
**2 (T2, PM, Pre)**	10	2.4 (1.0)		1.2 (0.7)	9	3.1 (1.5)		1.0 (0.5				
**2 (T2, PM, Post)**	10	71.8 (35.4)	32.2 (16.0)		10	62.9 (35.7)	25.3 (16.2					
**2 (T2, PM, Post 30)**	10	16.6 (27.0)	6.9 (5.7)		8	22.8 (14.8)	9.3 (7.0)					
**4**	8	3.0 (1.2)		1.4 (0.9)	9	3.4 (1.6)		1.4 (0.9	9	3.3 (1.6)		1.1 (0.4)
**7 (T3, AM, Pre)**	10	2.9 (1.9)		1.4 (1.3)	10	3.7 (1.7)		1.0 (0.3	10	3.6 (1.8)		1.2 (0.6)
**7 (T3, AM, Post)**	10	63.0 (39.2)	25.6 (9.8)		10	56.7 (29.2)	18.8 (7.8)					
**7 (T3, AM, Post 30)**	9	12.2 (13.2)	5.6 (3.6)		9	20.7 (12.2)	6.7 (2.8)					
**7(T3, PM, Pre)**	10	2.6 (1.0)		1.1 (0.4)	9	3.3 (2.1)		1.2 (0.8				
**7 (T3, PM, Post)**	10	67.1 (48.1)	27.1 (12.4)		10	61.6 (24.2)	25.0 (19.5)					
**7 (T3, PM, Post 30)**	10	20.3 (26.2)	7.2 (6.4)		10	23.6 (12.6)	8.3 (4.2)					
**+1**	10	2.5 (0.9)		1.2 (0.6)	10	3.8 (2.8)		1.2 (0.7	9	3.9 (1.9)		1.5 (1.3)
**+3 (T4, Pre)**	10	3.1 (1.4)		1.4 (0.9)	10	2.9 (1.7)		1.0 (0.3	10	4.0 (2.4)		1.6 (1.3)
**+3 (T4, Post)**	10	30.6 (12.0)	12.8 (7.2)		10	37.8 (21.0)	14.1 (9.3)		11	32.2 (15.1)	8.8 (3.2)	
**+7 (T5, Pre)**	9	3.2 (2.0)		1.5 (1.2)	9	2.7 (1.6)		1.0 (0.3	9	3.3 (1.2)		1.4 (0.8)
**+7 (T5, Post)**	10	30.4 (13.0)	11.8 (7.4)		10	34.5 (16.6)	12.2 (4.3)		10	29.3 (17.9)	11.0 (4.7)	
**+14 (T6, Pre)**	10	4.1 (2.8)		2.0 (1.6)	9	3.7 (2.6)		1.2 (0.5	11	4.0 (2.8)		1.8 (1.5)
**+14 (T6, Post)**	10	39.5 (13.6)	11.5 (6.3)		9	38.6 (20.0)	12.1 (2.4)		11	31.0 (14.0)	8.7 (5.0)	

cfDNA^{222}: circulating cell-free DNA concentration (ng/ml) measured by L1PA2 90 base pair assay and the mean acute and chronic fold changes are presented as mean (SD). FC^{acut}, acute fold changes of cfDNA^{222} based on Post/Pre or Post 30/Pre ratio for each time point. FC^{chronic}, chronic fold changes in cfDNA^{222} based on T0 Pre/T1 Pre, T0 Pre/T2 Pre ratio etc. High-intensity interval training shock microcycle (HSM), HSM with additional low-intensity training (HSM+LIT) and Control group (CG). Time point: time points during the study, AM and PM: sessions that took place in the morning (AM) and afternoon (PM), Pre, Post and Post 30: immediately before, after and 30 minutes after the exercise test., N: number of participants.
